# Impact of Different Oil Extraction Techniques on the Physicochemical Properties of *Adansonia digitata* Seed

**DOI:** 10.1155/2023/6233461

**Published:** 2023-10-25

**Authors:** Hayford Ofori, Ato Bart-Plange, Ahmad Addo, Komla Agbeko Dzisi

**Affiliations:** ^1^Department of Agricultural Engineering, Ho Technical University, P. O. Box HP 217, Ho, Ghana; ^2^Department of Agricultural and Biosystems Engineering, Kwame Nkrumah University of Science and Technology, Kumasi, Ghana

## Abstract

The seeds of baobab were found to have both industrial and domestic uses due to their essential oil qualities for topical medication. However, the seeds found in this study area in Ghana are underutilised and sometimes thrown away after being taken off the pulp. The present study is aimed at examining the impact of the two predominant techniques used for oil extraction from nonoily seeds, namely, mechanical extraction and Soxhlet (n-hexane) extraction, on both the oil yield and physicochemical properties of crude oil derived from baobab seeds. The study looked at the iodine value, peroxide value, acid value, colour, density, and other variables. Refractometers, chroma meters, and titration techniques were used for the determination of specific properties using standard methods. The Soxhlet method of oil extraction was superior in terms of maximum oil recovery, recording a value of 27.75%, in contrast to the mechanical method, which yielded a significantly lower recovery rate of 5.422%. The peroxide and iodine values were found to be 15.09 and 11.89 mEq/g and 85.89 and 88.45 g/100 g for the mechanical and Soxhlet extraction methods, respectively. Statistically significant differences (*p* ≤ 0.05) were observed between the two oil extraction methods in some of the properties measured. The study discussed the impact of these oil properties on the application of both food and nonfood products. Finally, the study has provided an essential set of data and information to enable product initiators in the cosmetic, food, and other industries to make informed decisions regarding the utilisation of baobab oil as a constituent in the formulation of products.

## 1. Introduction

Vegetable seed oil serves as a very beneficial energy source and facilitates cellular regeneration and repair in the human body. Seed oils have been used by many societies for several decades in various applications such as food, medicine, cosmetics, and biofuel. Oils and fats are integral constituents of dietary composition across various food categories, exerting a significant influence on sensory attributes such as flavour, quality, taste, and texture of food products. These factors are considered to be the fundamental constituents of food [1, 2]. The yearly global use of vegetable oil per capita is reported to be 18.15 kg, as stated in a report by the OECD/FAO [3]. Numerous food applications and enterprises exhibit a significant interest in plant-derived edible oils owing to their inherent natural qualities. The nutritional and caloric contents of fruit seeds make them valuable sources of edible oils and fats. Consequently, there has been a rise in demand for these oils, both in industrial applications and for human consumption [4].

Oilseeds significantly provide lipids for both human consumption and industrial use, exhibiting a notably higher oil concentration. Groundnut, soybean, palm kernel, cotton, olive, sunflower, rapeseed, sesame, linseed, and safflower, among other conventional oil seeds, have substantial oil content [5, 6]. Numerous plant seeds possess oils that are being employed to a significant extent within the beauty and pharmaceutical sectors. The search for novel natural constituents for application in the food, cosmetic, and pharmaceutical sectors has experienced a resurgence in recent times. In this regard, baobab (*Adansonia digitata* L.) seed oil has emerged as a subject of considerable investigation.

Baobab seed oil is an essential oil product that has had a remarkable surge in international markets. It has gained significant recognition in the domain of ethnobotany and ethnopharmacology, mostly due to its notable beauty-enhancing properties. Baobab seed oil, when applied topically, provides nourishing, moisturising, and occlusive properties, making it a valuable functional ingredient in the development of cosmetic formulations [7]. The utilisation of baobab seed oil is prevalent in the cosmetic sector, with its international distribution well established [8]. The baobab seed oil consists of approximately 33% oleic and linoleic acids [7]. Scientific literature has established that diets rich in linoleic and oleic acids can enhance the softness, replenishment, and moisturisation of the skin's outermost layer, known as the epidermis. In addition, the regenerative properties of the fatty acids found in baobab seed oil have been observed to stimulate the growth and renewal of epithelial tissues. This characteristic renders baobab seed oil very important within the cosmetic industry [9, 10]. Also, it is employed as a moisturiser for conditioning hair and nails and addressing the issue of dandruff. In addition to its primary applications, it is worth noting that baobab seed oil has been used in the manufacturing of lubricants, soaps, and toothpaste, as well as in the treatment of certain medical conditions such as muscular spasms, varicose veins, and wounds [10–12].

The global recognition and interest in the international marketing of baobab seed oil have increased in recent years, mostly due to its significant physicochemical properties and value [8]. Historically, the extraction of oil from oilseeds has been accomplished using various methods, including mechanical extraction (such as hydraulic and screw presses) that can be operated manually, semiautomatically, or fully automatically and solvent extraction. Additionally, a combination of two or more of these techniques has been used to extract oil from oilseeds. Currently, a diverse range of methodologies are employed for the extraction of oil from vegetable sources.

The extraction of oil from seeds is commonly achieved by mechanical means, employing electric screw press [13]. This method is considered the most ancient and widely recognised technique for extracting vegetable oils from the kernels of seeds [14, 15]. The use of mechanical oil extractor is predominantly observed in the extraction of seed oil, primarily owing to their convenient operational procedures and maintenance requirements [16]. Also, the oil obtained by using a screw press is free from any solvent contaminants, unlike the oil obtained using solvent extraction [17]. The multiple extractions of oil from the seeds using the expeller result in a higher extraction rate [18]. Nevertheless, the efficiency of oil recovery by using the mechanical screw press technique is low [19]. The efficiency of this procedure is compromised due to the presence of residual oil in both the cake and residue materials, which typically ranges from 8% to 14% [20]. Based on the findings of Willems et al. [19], it was observed that the inclusion of the shell or husk together with the seeds had a notable impact on oil yield, as it resulted in the absorption of a certain amount of oil during the extraction process.

Oil recovery from seeds using chemical or solvent extraction (Soxhlet) has proven to be effective [21]. Nevertheless, the use of these chemicals may result in oil contamination, causing harm to the environment. Additionally, the requirement for sophisticated equipment and proficient employees imposes constraints on its use [22]. Despite these restrictions, several researchers have employed the solvent extraction technique to extract seed oils [23, 24].

The demand for African baobab seed oil from European, Asian, and North American markets has experienced a significant surge in recent years. According to the Global Market Insights Report [25], the estimated worth of baobab products, specifically pulp and oil, was approximately US $3.7 billion in 2017. It is projected that this demand will increase to US $5.6 billion by the year 2024. The African countries exporting baobab seed oil to specialised markets include South Africa, Sudan, Tanzania, and Zimbabwe, as well as other states in West Africa [26, 27]. According to Donkor et al. [28], using baobab seed oil as food and nutraceutical items in Ghana can be beneficial for promoting health, mostly due to the oil's antioxidant properties. Zimbabwe, on an annual basis, exports approximately 20,000 litres of baobab oil with a market value of $100,000 [29].

Despite the global recognition and numerous studies conducted on the physicochemical properties of baobab seed oil in various countries such as Tanzania, Sudan, and Nigeria, there is a paucity of information in the literature regarding the baobab seed oil specifically found in the semideciduous agroecological zone of the Volta Region in Ghana. The seeds of baobab found in this agroecological zone of the Volta Region in Ghana are underutilised and neglected. The seeds are discarded after the pulp is separated from them. This could possibly be attributed to a lack of relevant data or information about the physicochemical composition of baobab seeds and their prospective applications. Research has demonstrated that the nutritional, antioxidant, and mineral composition of food components can be affected by the processing method employed. To efficiently extract and market baobab seed oil in this area, it is crucial to use the most appropriate processing methods that ensure optimal oil recovery and product quality. To this end, the empirical data about the physicochemical properties of baobab seed oil, specifically in relation to the two most common extraction methods, will provide baseline information for the advancement of the extraction and usage of baobab seed oil in this area. The acceptance and adoption of a prospective product may encounter setbacks if there is an insufficient amount of documented data and information available to substantiate its inherent features.

Therefore, the study was done to provide product developers in the food, cosmetic, and other industrial sectors who may be interested in baobab seed oil with a plausible baseline of information on the inherent properties of the oil found in this area of Ghana. The study is further aimed at assessing how the extraction techniques affect the physicochemical properties of baobab seed oil found in this area.

## 2. Materials and Methods

The present study employed a standardised approach to the materials and methods.

The baobab fruits were sourced from the semideciduous agroecological zone in the Volta Region of Ghana. The baobab pods were cracked using a machete, following which the contents were extracted and transferred into a receptacle. The seeds, together with the pulp, were pounded using a pestle and mortar to facilitate the separation of the pulp and fibre from the seeds. The contents followed sieving, wherein a 750 *μ*mm wire mesh sieve was employed to separate the pulp from the seeds and fibre. Subsequently, the seeds were washed to remove any residual pulp, followed by a period of sun-drying.

### 2.1. Methods of Oil Extraction

#### 2.1.1. Soxhlet Extraction

This is a widely used technique in analytical chemistry for the extraction of organic compounds from solid samples.

The seeds were subjected to pulverisation using a hammer mill and afterwards sieved through a 450 *μ*m screen to get smaller particle sizes. A total of 2,000 grams of baobab seeds was used, with 450 grams of milled-sieved samples used for the Soxhlet extraction process. The oil extraction process was conducted within the confines of the Chemistry Laboratory at the Kwame Nkrumah University of Science and Technology (KNUST). The extraction of seed oil was performed using n-hexane, an analytical grade solvent, at a temperature of 60°C. The milled seed was placed into a thimble within the Soxhlet apparatus, and the lower portion of the equipment was connected to a round bottom flask with a capacity of 1000 ml. A volume of 600 ml of hexane was introduced into the flask, and the upper section of the Soxhlet apparatus was equipped with a condenser. The heating mantle was used to maintain a consistent heat level, while the extraction process was conducted for a duration of up to 6 hours. The solvent was subjected to evaporation using a rotary evaporator, followed by subsequent drying in an open-air environment within a dark location. The oil yield was determined and subsequently transferred into airtight opaque containers, which were then stored in a refrigerated environment for subsequent analysis.

#### 2.1.2. Mechanical Extraction

**Figure 1:**
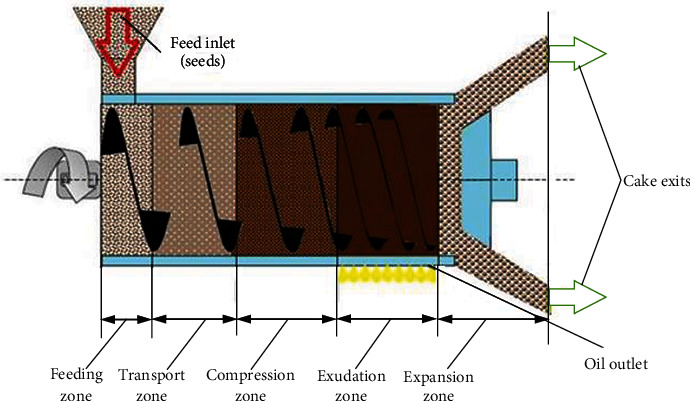
Schematic diagram of the mechanical extraction (adapted from Savoire et al. [30]).

The mechanical extraction of baobab seed oil was conducted at the workshop of the Department of Agricultural and Biosystems Engineering, Kwame Nkrumah University of Science and Technology, Kumasi. The schematic diagram shown in Figure 1 illustrates the mechanical extractor equipment employed in baobab seed oil extraction. The Reinartz model AP15 screw press for cold pressing was used, with a capacity of 750 kg/h. The extraction of the oil was done using the raw baobab seeds at an initial moisture content of 5.4% on a dry basis. The temperature of the extractor during the extraction process increased from 295 to 263 K, and the rate of extraction was estimated at 0.002 kg/s.

### 2.2. Determination of Oil Yield

The oil yield was calculated from the weight of extracted oil to the initial weight of the sample using

(1) $$\begin{array}{c} \text{Oil yield }\left(\%\right)=\frac{W_{\text{oe}}}{W_{\text{s}}}\times 100\%, \end{array}$$

where *W*_oe_ is the weight of extracted oil (g) and *W*_s_ is the initial weight of sample (g) before extraction.

### 2.3. Determination of Oil Physical Properties

The physical properties of baobab seed oil were assessed using the established methodologies recommended by the Japan Oil Chemists' Society (JOCS) [31].

#### 2.3.1. Density

The method prescribed by the Japan Oil Chemists' Society (JOCS, 2.2.2) was used for the determination of the density of the oil. A similar method adopted by Msalilwa et al. [32] was used for the determination of the density. This was carried out at room temperature. A known mass of a graduated measuring cylinder was used. A quantity of the oil was poured into the measuring cylinder, and the volume was read and recorded. The measuring cylinder containing the oil was weighed and the mass was recorded. The density of the oil was computed as in

(2) $$\begin{array}{c} \text{Density of oil }\left(\text{g}/\text{c}{\text{m}}^{3}\right)=\frac{M_{2}-M_{1}}{V}, \end{array}$$

where *M*_2_ is the mass of the cylinder and oil (g), *M*_1_ is the initial mass of the cylinder (g), and *V* is the volume occupied by oil in the cylinder (cm^3^).

#### 2.3.2. Refractive Index (RI)

The RI of the oil is the velocity of light in a vacuum to the velocity of light in the oil. The RI is a factor that determines the presence of fatty acids in the oil. It was measured using the standard method prescribed by the Japan Oil Chemist's Society (JOCS) (JOCS, 2.2.3). The butyrorefractometer was used. The instrument was initially calibrated using distilled water as a reference point at a temperature of 20°C. The sample was heated to make sure it was liquid and filtered. Two drops of oil were put on the lower prism and covered with the upper prism. It was held for one minute, and the reading was taken by looking through the end of the glass of the refractometer. The reading from the refractometer was converted to refractive index using conversion tables.

#### 2.3.3. Oil Colour

**Figure 2:**
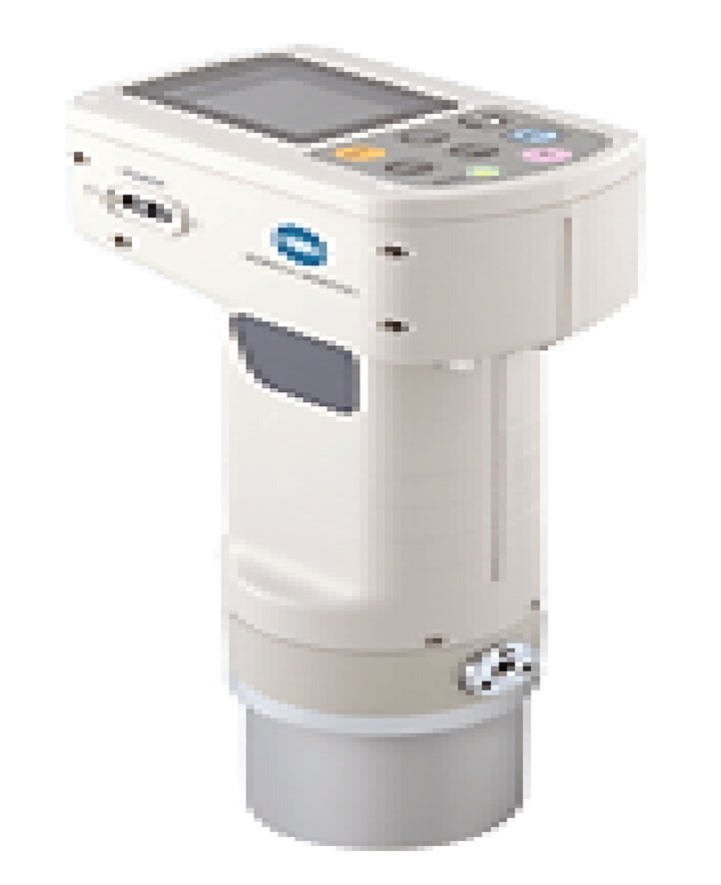
Chroma meter CR-410.

The colour of the baobab seed oil was determined using the chroma meter CR-410 colourimeter, a Konica Minolta, Inc. model. The device was set to read on different scale levels, that is, *Lab* and *L*^∗^*a*^∗^*b*^∗^, respectively. A tristimulus chroma meter measures colours using filters that match the cone cells in the human eye. Under standardised conditions, the system visually matches the colour against the three primary colours, which are red, green, and blue. The three results are expressed as *X*, *Y*, and *Z*, which are translated as *Lab* and *L*^∗^*a*^∗^*b*^∗^ on the Hunter's and CIELAB scales, respectively, in this study.  Figure 2 shows the chroma meter used for measuring the colour of the baobab seed oil. The JOCS (2.2.1) standard method was used to measure the colour of the oil as a factor of the presence of chlorophyll pigment in the oil.

#### 2.3.4. Boiling and Melting Points of the Oil

A similar method described by Idris et al. [24] was used to determine the boiling (JOCS, 2.2.11) and melting points (JOCS, 2.2.4) of the oil. A thermometer was placed inside a glass vial containing 10 ml of oil. The oil in the glass vial was brought on a mantle with low heat. The temperature was measured and noted as the boiling temperature when the oil started to boil. A glass vial with a thermometer was filled with 10 ml of the oil. The oil in the glass vial was put into a container with ice cubes to solidify. The oil's melting point is when it is heated in a water bath to a temperature higher than room temperature to melt after solidification.

### 2.4. Determination of Oil Chemical Properties

The chemical analysis of the baobab seed oil was conducted using the established procedures recommended by the Japan Oil Chemists' Society (JOCS) [31]. Each property was assessed using the corresponding standard method as specified.

#### 2.4.1. Iodine Value (JOCS, 2.3.4)

A similar method adopted by El-Arab [33] was used for the determination of the iodine value. The IV is a measure of the average number of double bonds of oil or fat. In this method, 0.2 g of baobab oil was dissolved in cyclohexane, and 0.1 M iodine acetic acid was added. The mixture was held for 20 minutes to allow for halogenation. To free iodine and reduce excess iodine monochloride, 0.1 M of potassium iodide solution was added. The freed iodine was titrated with a standard solution of 0.1 M sodium thiosulphate using a starch indicator. Equation (3) was used to determine the iodine value.

(3) $$\begin{array}{c} \text{IV }\left(\text{g}/100\text{ g}\right)=\left\{\frac{\left[\left(B-S\right)\times M\times 12.69\right]}{\text{Sample weight}}\right\}, \end{array}$$

where *B* is the blank titre value, *S* is the sample titre value, *M* is the molarity of sodium thiosulphate, and 12.69 is the conversion factor.

#### 2.4.2. Peroxide Value (JOCS, 2.5.2)

The peroxide value of a fat reflects the degree of its oxidation taking place, and it also determines the shelf life of the oil. The greater the PV, the shorter the shelf life [32]. A similar method used by Idris et al. [24] was adopted. Two grams of the sample was put into a 250 ml stoppered conical flask. A solvent mixture of acetic acid-chloroform (30 ml) was added and the mixture swirled to dissolve. 0.5 ml of saturated potassium iodide solution was added and occasionally shaken within one minute after allowing it to stand, and 30 ml of distilled water was added. The obtained mixture was titrated against the liberated iodine of 0.1 N sodium thiosulphate solution; during the process, it was shaken vigorously until the yellow colour disappeared. 0.5 ml of starch solution (as an indicator) was added and titration continued while shaken vigorously until a blue colour disappeared. The peroxide value (PV) was determined using

(4) $$\begin{array}{c} \text{PV }\left(\text{mEq}/\text{g}\right)=\frac{S_{\text{t}}-M_{\text{s}}}{S_{\text{w}}}\times 1000, \end{array}$$

where *S*_t_ is the titration of standard (ml), *M*_s_ is the molarity of standard (M), and *S*_w_ is the weight of the sample (g).

#### 2.4.3. Saponification Value (JOCS, 2.4.2)

Is the amount of alkali (in the case of magnesium or potassium hydroxide) saponify a definite quantity of fat or oil? For this analysis, one gram of the baobab oil was put into a 250 ml conical flask. 15 ml of potassium hydroxide was pipetted and added, and the content was refluxed for one hour. Simultaneously, a blank was prepared in another conical flask containing 1.0 g of distilled water. Using a standardized 0.1 M hydrogen chloride and phenolphthalein as an indicator, the sample was titrated until a pink colour was reached. The volume of the hydrogen chloride (HCl) was recorded, and the saponification value (SV) was determined using [32]

(5) $$\begin{array}{c} \text{SV }\left(\text{mg KOH}/\text{gof sample}\right)=\frac{\left[\left(B-S\right)\times N\times \left(0.5\times 56.1\right)\right]}{\text{weight of sample}}, \end{array}$$

where *B* is the solution required for the titration of the blank (ml), *S* is the solution required for the titration of the sample, *N* is the normality of the HCl solution, and 56.1 is the molecular weight of KOH.

#### 2.4.4. Acid Value (JOCS, 2.3.1)

The acid value of the oil was measured using JOCS, 2.3.1, standard method based on titration. A sample of crude oil that had been homogenized and sieved weighing about 3 grams was put into a 250 ml conical flask. Following the addition of 1 ml of phenolphthalein indicator, 50-100 ml of freshly neutralized, hot ethyl alcohol was added to the oil sample. The mixture was heated for about fifteen minutes in a water bath and then titrated against a standard 0.1 N potassium hydroxide solution in a hot state until the mixture developed a pink colour that lasted for about 20 seconds. Equation (6) was used to determine the acid value.

(6) $$\begin{array}{c} \text{Acid value }\left(\text{mg KOH}/\text{g}\right)=\frac{\left(V\times N\right)\times 56.1}{W}, \end{array}$$

where *V* is the volume in ml of standard potassium hydroxide, *N* is the normality of the potassium hydroxide solution, and *W* is the weight of the sample (g).

### 2.5. Statistical Analysis

The statistical analysis was carried out using RStudio (version 2022.12.0 Build 353) and Microsoft Excel. Each parameter was replicated three times, and the results were presented as means ± SD. The data were subjected to a one-way analysis of variance (ANOVA). Tukey's HSD post hoc test was performed to evaluate the significant differences among the means at the 5% significance level.

## 3. Results and Discussion

### 3.1. Oil Yield

The physicochemical properties of the baobab seed oil using mechanical and Soxhlet extractions (n-hexane) are shown in Table 1. The oil recovery rate was higher in the Soxhlet extraction than what was recovered in the mechanical extraction. The oil yield in the mechanical extraction for baobab seed was found to be 5.422%, and that of the Soxhlet extraction was 27.75%. Cissé et al. [23] found the oil yield of baobab seed from the Bignona community in Senegal at 6.28% and 30.29%, respectively, for cold pressing and n-hexane extraction. Idris et al. [24] also found 33.83% oil content of Sudanese baobab seeds. Also, Abubakar et al. [34] found the oil content of baobab seed at 32.0% from the Jigawa state in Nigeria. More so, the oil content from Adamawa state in Nigeria was found to be 45.0% [12]. Though the oil content in baobab seed found in this study was lower than what was reported by researchers from other countries, it is consistent with the average oil yield reported in the literature, which states that most plant seeds contain about 22-45% of oil on dry matter basis [24]. The lower content and differences in the oil yield may be due to varietal differences in the baobab seeds and other pretreatments of the seed before extraction.

### 3.2. Density of the Oil

The densities of the baobab seed oil were found to be 0.87 g/cm^3^ and 0.97 g/cm^3^, respectively, for mechanical extraction and Soxhlet extraction (shown in Table 1). According to Msalilwa et al. [32], the purity of the oil is determined by its density or specific gravity. They posit that the presence of a higher value of fatty acid found in the oil increases the specific gravity or density of the oil. They found the specific gravity of baobab seed oil to be 0.928. Cissé et al. [23] found that the densities of baobab seed oil were 0.911 and 0.902 g/cm^3^, respectively, for pressure and n-hexane extractions. The density of baobab seed oil found in this study agrees with what is reported in the literature. No significant difference was observed in the densities of the baobab seed oil with the use of the two extraction methods in this study.

### 3.3. Melting and Boiling Point Temperatures and Refractive Index

In Table 1, the melting and boiling temperatures of baobab crude oil were found to be 10.67 and 12.3°C and 225 and 230.3°C, respectively, for mechanical extraction (ME) and Soxhlet extraction (SE). No significant difference was found between ME and SE at *p* ≤ 0.05. The refractive index (RI) for ME and SE was determined at 1.425 and 1.431, respectively. The RI of baobab crude oil is a reflection of the presence of unsaturated fatty acids [24]. Idris et al. [24] and Nkafamiya et al. [12] found the RI of baobab oil at 1.436 and 1.459, respectively. The RI by Codex standard for peanut oil, maize oil, and palm oil were found to be in a range of 1.460–1.465, 1.465–1.468, and 1.454–1.456, respectively [35]. The refractive index is also a determinant factor of oil rancidity. The higher the RI, the more the oil is prone to rancidity [36]. The RI found in this study for baobab seed oil, though lower than what was obtained for peanut, maize, and palm oils, is within the range that the oil found in this study may not be extremely prone to rancidity or decay.

### 3.4. Peroxide Value

The peroxide value (PV) of the oil is a quality determinant that indicates the oxidative nature of the oil. PV is used to monitor the oxidative degradation of oils and their rancidity [37]. The value of PV for ME was higher at 15.09 mEq/g than that of the SE, which was found to be 11.89 mEq/g in this study (Table 1). A significant difference was observed in the PV for ME and SE. The PV registered in this study is consistent with the PV of 13.5 found by Affo and Akande [38]. Idris et al. [24] and Ndiaye et al. [39] found lower peroxide values of 4.3 mEqO2/kg and 0.5 mEqO2/kg, respectively. Although the higher PV reported in this study suggests that the oil is healthier to use and has a larger proportion of unsaturated fatty acids, the higher PV is an indication of the oil's susceptibility to oxidation and quality degradation during processing and storage [40].

### 3.5. Acid Value

The acid value (AV) of vegetable oil is an indication of the edibility of the oil, and it further determines the amount of free fatty acid in fats and oils. According to FAO/WHO recommendation (AOCS Official Method Cd 8-53, 2003), the permissible level of AV for all edible oils should be below 0.6 mg KOH/g. The AV (Table 1) were found to be 9.31 and 4.57 mg KOH/g for mechanical extraction and Soxhlet extraction, respectively. Affo and Akande [38] found AV in baobab seed oil at 6.8 mg KOH/g. Also, Cissé et al. [23] found baobab seed oil to contain an AV of 18.827 and 12.442 mg KOH/g, respectively, for using pressure and n-hexane extractions. The AV found in this study is higher than the permitted AV for edible oils, which is less than 4 mg KOH/g [41]. To make this oil edible, further processing is recommended to reduce the AV.

### 3.6. Saponification Value

The most popular quality metric for evaluating oils and fats is saponification value (SV). It is the quantity of alkali (reported as mg KOH/g sample) needed to saponify a specific amount of sample fat (1 g fat) [42]. The length of the triacylglycerol's fatty acyl chains affects SV (the major form of dietary lipid in fats and oils). The lower the fatty acyl chain, the lower the triglycerides, and the higher the SV. In Table 1, the SVs for baobab seed oil in this study were 175.2 mg KOH/g for mechanical extraction and 179.9 mg KOH/g for n-hexane Soxhlet extraction. No significant difference was observed for ME and SE at *p* ≤ 0.05. Cissé et al. [23] observed SV at 233.587 and 209.198 mg KOH/g for pressure and n-hexane extractions, respectively. Additionally, Nkafamiya et al. [12] used petroleum ether as an extractant and found baobab seed oil to contain SV of 196 mg/KOH. A study conducted by Bhuiya et al. [21] found that the SVs of beauty leaf (*Calophyllum inophyllum* L.) oil extracted mechanically and chemically (using n-hexane) were 192.2 mg/g and 192.0 mg/g, respectively. Saponification values determined by Codex standards for coconut oil, cotton seed oil, and palm kernel oil were found in the range of 248-265, 189-198, and 230–254, respectively [35]. The SV found in this study is lower compared to what is reported in the literature for cotton seed, sunflower, soybean, and rapeseed oils, whose saponification values are reported in a range of 168–196 mg KOH/g oil [43]. SV ranging from 235 to 260 mg KOH/g oil is considered high [43]. The lower SV in this study is a sign that the baobab oil glycerol backbone contains long-chain fatty acids [42] making it an important energy source for many tissues.

### 3.7. Iodine Value

One of the quality attributes used to evaluate oil is the iodine value. It is a measurement of the fatty acid's unsaturation and is used to assess the susceptibility of the oil to oxidation [44]. The iodine value (IV) for mechanical extraction and Soxhlet extraction in this study as in Table 1 was found to be 85.89 and 88.45 g/100 g, respectively. There was no significant difference in the IV for both mechanical and chemical extractions (*p* ≤ 0.05). According to Tsado et al. [4], oils with an IV less than 100 ml/g are referred to as nondrying oils. In this study, the IV of the baobab seed oil which is <100 g/100 g for both extraction methods cannot be used in the production of ink and paint due to its nondrying characteristics. The results are comparable to those obtained by Nkafamiya et al. [12] for baobab seed oil from Adamawa state in Nigeria who found IV of 87 g/100 g. El-Arab [33] of Sudan (Kosti, city) found an IV of 86 g/100 g for baobab seed oil. Baobab seed oil from the Bignona region in Senegal was found to have iodine values of 99.113 and 90.775 mg/100 g when it was extracted under pressure and with n-hexane, respectively [23]. Additionally, similar results were obtained for IV for olive and peanut oils in the range of 74-88 and 84-100 g/100 g, respectively [45]. For the manufacturing of cream made with vegetable oil, an oil and fat with an iodine value of at least 150 mg/100 g is preferable [46].

### 3.8. Oil Colour

According to Balbino et al. [47], the colour of crude oil is an important determinant factor of market value. In this study, the oil colour was analysed and presented on two scales, as shown in Table 2. The expressed colour contents of the baobab seed oil are based on the Hunter (*Lab*) and CIELAB (*L*^∗^*a*^∗^*b*^∗^) scales. The *L*/*L*^∗^ shows that the baobab seed oil for both methods were lighter in colour; that is, both values exceeded 51 points on the scale of 51–100 as in Islam et al. [48] and Dodoo et al. [49]. The values of *L*/*L*^∗^ for the mechanical and Soxhlet extractions were found to be 61.99/68.37 and 69.70/72.19, respectively. On the Hunter's scale, there was no significant difference for *L* at *p* ≤ 0.05, but a significant difference was observed for *L*^∗^ on the CIELAB scale. The values of *b*/*b*^∗^ were 35.65/59.34 and 38.14/66.83, respectively, for mechanical and Soxhlet extractions. Both values were found to be positive, which is an indication of the yellow colour of the baobab seed oil. Significant differences were observed for *b*/*b*^∗^ both on the Hunter and CIELAB scales. Also, the values on the scale for the *a*/*a*^∗^ were both positive for the two methods used in the oil extraction. The values were 2.15/2.34 and 2.53/2.75 for mechanical and Soxhlet extractions, respectively. The positive colour on the *a* scale is an indication of a reddish colour. From the analysis, the baobab seed oil for both methods of extraction in this study can be said to be bright or light yellowish red in colour. This is due to the dominance of lightness (*L*/*L*^∗^), yellow (*b*/*b*^∗^), and red (*a*/*a*^∗^) in the colour of the oil. Idris et al. [24] also found baobab seed oil to be reddish yellow at 25°C. Abubakar et al. [34] reported that the baobab seed oil is light yellow in colour. Cissé et al. [23] found different colours for different solvents used for baobab seed oil extraction, and they attribute it to the polarity of the solvents used. The quantity of chlorophyll found in the baobab seeds from different ecological zones may account for the varying colours of the oil [50]. According to Orhevba et al. [51], moisture content (MC) has an impact on colour intensity. That is, an increase in MC raises the level of chlorophyll, which raises the colour level.

## 4. Conclusion

The results found in this study suggest that the oil yield was higher in the Soxhlet extraction (27.75%) than that of the mechanical extraction (5.422%). The refractive index was found to be 1.425 and 1.431 for mechanical and Soxhlet extractions, respectively. The higher peroxide values obtained in this study are an indication of the presence of unsaturated fatty acids in the oil. This makes the oil healthier for use. However, the high peroxide value found in this study makes the oil susceptible to oxidation and deterioration during processing and storage. Also, the high acid value in the oil signifies the unsuitability of the oil for edible purposes. Though the value obtained for the iodine value of baobab seed oil in this study cannot be used in ink and paint production due to its nondrying characteristics, the value nonetheless suggests its usage in the manufacture of soap and ice creams made with vegetable oils. The study has revealed that the baobab seed oil found in this study area possesses some essential physicochemical properties that can be harnessed for use in the improvement of human existence. Research using different extraction methods can be employed to assess the physicochemical properties of the oil. In addition, the fatty acid profile, antioxidants, and *p*-anisidine values of the oil are worth assessing.

## Figures and Tables

**Table 1:** Physicochemical properties of baobab seed oil.

Parameter	Mechanical extraction	Soxhlet extraction	LSD
*Physical properties*			
Oil yield (%)	5.422	27.75	—
Density (g/cm^3^)	0.87b ± 0.0	0.97a ± 0.02	0.04
Melting point (°C)	10.67a ± 1.5	12.3a ± 0.6	ns
Boiling point (°C)	225b ± 2	230.3a ± 2.1	4.63
Refractive index	1.425a ± 0.0	1.431a ± 0.0	ns
*Chemical properties*			
Peroxide value (mEq/g)	15.09a ± 0.3	11.89b ± 0.2	0.59
Acid value (mg KOH/g)	9.31a ± 0.1	4.57b ± 0.3	0.54
Saponification value (mg KOH/g)	175.2a ± 2.5	179.9a ± 2.9	ns
Iodine (g/100 g)	85.89a ± 1.2	88.45a ± 2.1	ns

Values are means of triplicate ± standard deviation. Means followed by the same letter in the same row are significantly not different at *p* ≤ 0.05.

**Table 2:** The baobab seed oil colour contents using mechanical extraction and Soxhlet extraction.

Colour						
	Hunter scale (*Lab*)			CIELAB scale (*L*^∗^*a*^∗^*b*^∗^)		
Extraction method	*L*	*a*	*b*	*L* ^∗^	*a* ^∗^	*b* ^∗^
Mechanical	61.99b ± 1.5	2.15 ± 0.4	35.65b ± 0.3	68.37 ± 1.4	2.34 ± 0.4	59.35b ± 1.2
Chemical (Soxhlet)	69.7a ± 1.3	2.53 ± 0.5	38.14a ± 0.4	72.19 ± 2.3	2.75 ± 0.6	66.83a ± 1.8
MSD	3.2	ns	0.8	ns	ns	3.4

Values are means of triplicate ± standard deviation. Means followed by the same letter in the same column are significantly not different at *p* ≤ 0.05. Values of *L*/*L*^∗^ ≥ 51 are an indication of lightness, and values ≤ 50 are blackness. Positive values of *b*/*b*^∗^ are an indication of yellowish colour, and negative values are an indication of blueness. Positive *a*/*a*^∗^ values indicate a reddish colour, while negative values indicate a greenish colour.

## Data Availability

The primary data used to support the findings of this study are available from the corresponding author upon request.
